# EFEMP1 Overexpression Contributes to Neovascularization in Age-Related Macular Degeneration

**DOI:** 10.3389/fphar.2020.547436

**Published:** 2021-01-15

**Authors:** Lu Cheng, Chong Chen, Wenke Guo, Kun Liu, Qianqian Zhao, Ping Lu, Fudong Yu, Xun Xu

**Affiliations:** ^1^ Shanghai Key Laboratory of Ocular Fundus Diseases, Department of Ophthalmology, Shanghai General Hospital, Shanghai Jiaotong University, National Clinical Research Center for Eye Diseases, Shanghai Engineering Center for Visual Science and Photomedicine, Shanghai Engineering Center for Precise Diagnosis and Treatment of Eye Diseases, Shanghai, China; ^2^ NHC Key Lab of Reproduction Regulation, Shanghai Institute of Planned Parenthood Research, Fudan University, Shanghai, China

**Keywords:** Age-related macular degeneration, EFEMP1, fibulin-3, choroidal neovascularization, biomarker, gene expression profiling

## Abstract

**Purpose:** Age-related macular degeneration (AMD) is one of the leading causes of blindness, and choroidal neovascularization (CNV) in AMD can lead to serious visual impairment. Gene expression profiling of human ocular tissues have a great potential to reveal the pathophysiology of AMD. This study aimed to identify novel molecular biomarkers and gene expression signatures of AMD.

**Methods:** We analyzed transcriptome profiles in retinal-choroid tissues derived from donor patients with AMD in comparison with those from healthy controls using a publicly available dataset (GSE29801). We focused on the EFEMP1 gene, which was found to be differentially upregulated in AMD, especially in wet AMD eyes. Serological validation analysis was carried out to verify the expression of EFEMP1 in 39 wet AMD patients and 39 age- and gender-matched cataract controls, using an enzyme-linked immunosorbent assay (ELISA). We then investigated the role of EFEMP1 in angiogenesis through *in vitro* experiments involving EFEMP1 overexpression (OE) and knockdown in human umbilical vein endothelial cells (HUVECs).

**Results:** An increase in EFEMP1 expression was observed in the retinal-choroid tissues of eyes with AMD, which was more significant in wet AMD than in dry AMD. In addition, there was a significant increase in serum fibulin-3 (EFEMP1 encoded protein) concentration in patients with wet AMD compared with that in the controls. Tube formation and proliferation of EFEMP1-OE HUVECs increased significantly, whereas those of EFEMP1 knockdown HUVECs decreased significantly compared with those of the control. Additional extracellular fibulin-3 treatments did not increase tube formation and proliferation of wildtype and EFEMP1 knockdown HUVECs, indicating that the proangiogenic properties of EFEMP1 are of cell origin. We also found that vascular endothelial growth factor expression in HUVECs was upregulated by EFEMP1 overexpression and downregulated by EFEMP1 knockdown.

**Conclusion:** Our findings demonstrate EFEMP1 as a novel biomarker for CNV in AMD, providing a new target for the development of wet AMD-directed pharmaceuticals and diagnostics.

## Introduction

Age-related macular degeneration (AMD) is one of the leading causes of blindness in patients beyond 55 years. The number of AMD-affected patients is predicted to reach nearly 200 million by 2020 globally, increasing to approximately 300 million by 2040 (Wong et al., 2014). In advanced cases, wet AMD is often associated with abnormal choroidal neovascularization (CNV), a phenotype that can cause fluid and lipid leakage under the macula and fibrous scar formation and ultimately lead to serious visual impairment (Schmidt-Erfurth and Waldstein, 2016).

Although it is well-known that aging is the prevailing risk factor for AMD, genetic factors may contribute to AMD occurrence and progression (Smith et al., 2001). In recent years, genetic linkage analysis and genome-wide association studies have identified several important genetic risk factors, including complement-related genes (CFH, C2, CFB, CFHR1/3, C3, etc.) as well as non-complement-related genes, such as ARMS2 and HTRA1, and lipid metabolism-related loci (Zareparsi et al., 2004; Edwards et al., 2005; Hageman et al., 2005; Gold et al., 2006; Yang et al., 2006; Maller et al., 2007; Fritsche et al., 2016). Despite these important discoveries, we still lack a detailed insight into the molecular mechanism responsible for the specific AMD phenotype. Although the introduction of treatments targeting vascular endothelial growth factor (VEGF) has decreased the incidence of legal blindness and visual impairment caused by wet AMD (Mehta et al., 2018), the underlying CNV pathophysiology and a comprehensive understanding of the biological pathways that mediate wet AMD development and progression have not yet been clarified.

Compared to research strategies that depend on indirect and reductionist experimental approaches, gene expression profiling of human ocular tissues has great potential to resolve AMD-associated molecular signaling pathways more precisely and comprehensively (Newman et al., 2012). Therefore, in this study, we analyzed the transcriptome profiles of differentially expressed genes in ocular tissues derived from AMD donor patients and compared the results with those of healthy donor controls using published public datasets and focused on the differentially upregulated epidermal growth factor-containing fibrillin-like extracellular matrix protein 1 (EFEMP1) gene. To elucidate the possible role of EFEMP1 in wet AMD and its biological function, serological validation analysis was carried out to verify the expression of EFEMP1 in wet AMD patients and cataract controls using enzyme-linked immunosorbent assay (ELISA). The phenotype was detected in EFEMP1 overexpressing and EFEMP1 knockdown human umbilical vein endothelial cells (HUVECs). Our findings might reveal a potential new target for the development of wet AMD-directed pharmaceuticals and diagnostics.

## Materials and Methods

### Screening Differentially Expressed Proteins Through Published Datasets

DNA-free RNA levels of differentially expressed proteins in retinal-choroid samples from human donor eyes were screened in two published datasets, the University of Iowa and the Lions Eye Bank of Oregon. Unlike the Iowa samples, which were expertly graded (normal, pre-AMD, dry AMD, and wet AMD), the Oregon samples received only a general AMD classification based on medical histories confirmed by ophthalmological records (Newman et al., 2012). Since Oregon samples received a less rigorous AMD classification than the Iowa samples, only normal and pre-AMD data were included. Donor-specific details (e.g., age, sex, and AMD phenotype) can be evaluated using the Gene Expression Omnibus (GEO: GSE29801).

### Patient Samples

This study was reviewed and approved by the Medical Ethics Committee at Shanghai General Hospital affiliated to Shanghai Jiao Tong University (No.2016KY115-2) and conformed to the tenets of the Declaration of Helsinki. Written informed consent was obtained from all participants.

Patients were screened for enrollment in Shanghai General Hospital from October 2018 to December 2018. Patients diagnosed with wet AMD were included, assessed independently by two trained ophthalmologists (Dr. Xun X and Dr. Kun L). Patients were excluded if 1) they had intraocular surgeries or other pathologies, including congenital ocular diseases, glaucoma, and fundus diseases except for AMD according to self-reported history or ophthalmic examination; 2) they had systematic diseases including liver damage, kidney failure, lung disease, mental illness, autoimmune diseases, or cancer; and 3) the participant was unwilling or unable to give written consent or verbal assent. After the enrollment, each participant underwent a comprehensive ophthalmic examination, including a best-corrected visual acuity (BCVA) evaluation, slit-lamp biomicroscopy, tonometry, fundus examination, and optical coherence tomography (OCT). BCVA was measured using a retroilluminated Early Treatment of Diabetic Retinopathy Study chart from a distance of 4 m. For the control group, we enrolled age- and gender-matched cataract patients without fundus diseases who planned to undergo cataract surgery at Shanghai General Hospital. All serum samples were collected from wet AMD patients and cataract controls and stored in a −80°C refrigerator for no more than three months after quick-freezing in liquid nitrogen.

### Serological Validation Analysis: ELISA-Based EFEMP1 Quantification

ELISA for EFEMP1 coding protein fibulin-3 was conducted according to the manufacturer’s instructions using the Human EFEMP1 Assay ELISA Kit (#SEF422Hu, USCN Life Science Inc., Wuhan, China). Absorbance was measured at 450 nm using a microplate reader (Model 680, Bio-Rad, Hercules, CA, United States), and the results were calculated using GraphPad PRISM 5.0 (GraphPad Software Inc., La Jolla, CA, United States). Log transformation was performed for all analyses. Fibulin-3 levels were calculated and expressed as ng/mg. All samples were tested in duplicate.

### Cells and Reagents

Primary HUVECs from pooled donors were obtained from Lonza (Portsmouth, NH) and maintained in endothelial cell medium (ECM, ScienCell, San Diego, CA, United States). To mimic the AMD-associated EFEMP1 transcriptional level increase, we constructed EFEMP1 overexpressing (EFEMP1-OE) HUVECs using a recombinant lentivirus vector GV492 containing the human EFEMP1 gene (GenBank: NM_002775; SIRION Biotech). The lentivirus vector for EFEMP1 knockdown was achieved by cloning small hairpin RNAs using a recombinant lentivirus vector GV248, described as shEFEMP1 (target sequence: 5′-gcG​TAG​ACA​TAG​ATG​AAT​GTA-3′). CON335 and CON077 modification-containing enzymatically inactive variants were used separately as controls for EFEMP1-OE and shEFEMP1, respectively. Green fluorescent protein (GFP) was also inserted into the vector to instantly monitor the transfection rate. After 2 weeks of culture, when the HUVEC monolayer was completely established, cells were infected with GV492-EFEMP1, GV492-CON335, GV248-shEFEMP1, and GV248-CON077 overnight at 37°C. The medium was then changed, and the cells were cultured for three more weeks before performing any experiment.

### Quantitative Real-Time Polymerase Chain Reaction

Total RNA was extracted using TRIzol (Invitrogen, Carlsbad, CA, United States), and reverse transcription was performed through M-MLV and cDNA amplification using the SYBR Green Master Mix kit (Takara, Otsu, Japan). Total RNA was isolated using a High Pure miRNA isolation kit (Roche) and the reverse transcription reaction was performed using a TaqMan MicroRNA Reverse Transcription kit (Life Technologies). Nuclear and cytoplasmic fractions were isolated using NE-PER Nuclear and Cytoplasmic Extraction Reagents (Thermo Scientific). Primer sequences are listed in Table 1. qRT-PCR results were analyzed using the LightCycler480 (Roche). The results shown represent the average of three independent experiments.

**TABLE 1:** Sequences of primers used in this study.

Gene Name	Direction	Sequence 5′ to 3′
EFEMP1	Forward	CAG​GCT​ACG​AGC​AAA​GTG​AAC
	Reverse	ACA​GTT​GAG​CCT​GTC​ACT​GCT
VEGF	Forward	CAA​CTT​CTG​GGC​TGT​TCT​CGC​T
	Reverse	CCC​CCT​CTC​CTC​TTC​CTT​CTC​T

### Western Blot Analysis

HUVECs were harvested and extracted using a lysis buffer (100 mM Tris-HCl, 2% SDS, 1 mM mercaptoethanol, 25% glycerol). Cell extracts were boiled in SDS sample buffer (Invitrogen, Carlsbad, CA, United States) and equal amounts of cell extracts were separated on 15% SDS-PAGE gels. Separated protein bands were transferred onto polyvinylidene fluoride membranes (Millipore, Billerica, MA, United States). The primary antibodies, including anti-fibulin-3 (ab70561, rabbit polyclonal antibody, Abcam, Cambridge, MA, United States), anti-VEGF (ab53465, rabbit polyclonal antibody, Abcam, Cambridge, MA, United States), and anti-tubulin (ab153802, rabbit polyclonal antibody, Abcam, Cambridge, MA, United States), were diluted at a ratio of 1:1,000 according to the manufacturer’s instructions and incubated overnight at 4°C. Horseradish peroxidase-linked secondary antibodies (Cell Signaling Technology) were added at a dilution ratio of 1:10,000 and incubated at room temperature for 1 h. The membranes were washed with PBS three times and the immunoreactive bands were visualized using ECL-PLUS/Kit (GE Healthcare, Piscataway, NJ, United States) according to the manufacturer’s instructions.

### Human Umbilical Vein Endothelial Cell Tube Formation Assay

We used a HUVEC tube formation assay, which measures the ability of endothelial cells plated at subconfluent densities with the appropriate extracellular matrix support, to form capillary-like structures (tubes) to model the reorganization stage of angiogenesis. Experiments were performed using a µ-Slide Angiogenesis kit (81,506, Ibidi, Martin Reid, Germany). Precooled growth factor-reduced Matrigel was added to each inner well of cooled µ-Slide plates and incubated at 37°C for 60 min. HUVECs were preincubated at 37°C for 30 min before seeding. In some cases, WT or shEFEMP1 HUVECs were preincubated in ECM with different concentrations of recombinant human fibulin-3 protein (8416-FB-050, R&D Systems, Minneapolis, MN). 50 µl cell suspension (2 × 10^5^ cells/ml) with or without fibulin-3 protein was applied to each upper well. After incubation at 37°C for 12 h, the samples were stained with diluted calcein (6.25 µg/ml), fluorescent images were captured using an Olympus FSX100 microscope (Olympus, Tokyo, Japan), and the images were analyzed using the ImageJ software (National Institutes of Health, Bethesda, MA, United States). Four fields were randomly selected from each well, then the total tube length, number of branch points, and total number of networks were quantified.

### Human Umbilical Vein Endothelial Cell 5-Ethynyl-2′-Deoxyuridine Proliferation Assay

HUVECs were seeded onto a 96-well plate at a density of 1 × 10^4^ cells per well in ECM with or without fibulin-3 protein and allowed to adhere overnight. 5-Ethynyl-20-deoxyuridine (EdU) assay was carried out with a Click-iT™ Plus EdU Cell Proliferation Kit (C10638, Thermo Fisher Scientific, Waltham, MA, United States). After incubation with 50 µM EdU for 2 h, the cells were fixed in 4% paraformaldehyde and stained with Apollo Dye Solution. Hoechst-33,342 was used to stain nucleic acids within the cells. Images were acquired with an Olympus FSX100 microscope (Olympus, Tokyo, Japan), and the percentage of EdU-positive cells was quantified using the ImageJ software (National Institutes of Health, Bethesda, MA, United States).

### Statistical Analyses

Results for continuous variables with normal distribution are presented as mean ± SD and were analyzed using one-way analysis of variance (ANOVA) and Student’s t-test. Bonferroni correction was used for multiple comparisons. Results for continuous but non-normally distributed variables were presented as medians and interquartile ranges and analyzed using nonparametric tests. Binary variables were presented as absolute numbers or percentages and were analyzed using the chi-squared test. Statistical analyses were performed using SPSS 22.0 (Statistical Software, Los Angeles, CA, United States) and GraphPad PRISM 5.0. *p*-values <0.05 were considered statistically significant.

## Results

### Identification of EFEMP1 in Age-Related Macular Degeneration

**FIGURE 1:**
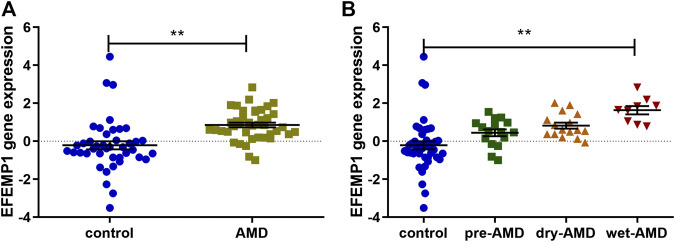
EFEMP1 gene expression in ocular tissues derived from patients with age-related macular degeneration (AMD) and healthy controls from GSE29801. **(A)** EFEMP1 expression increased in AMD-affected eyes compared with that in the control group. **(B)** EFEMP1 expression in wet AMD-affected eyes was higher than that in the control group as well as in the pre-AMD and dry AMD groups. **p < 0.01.

To identify the differentially expressed genes in AMD, we performed transcriptome profiling from GSE29801 (containing retinal-choroid tissue samples from 42 normal eyes and 41 eyes with AMD, including 16 pre-AMD eyes, 16 dry AMD eyes, and nine wet AMD eyes) after data preprocessing and quality assessment using R software. We identified 827 upregulated genes and 592 downregulated genes; the top 100 most significant DEGs of GSE29801 have been identified in another study of ours (not published yet). Furthermore, tissue specific expression analysis of these DEGs was conducted to obtain retinal-choroid specific proteins. EFEMP1/fibulin-3, the expression of which is significantly higher in retinal-choroid than in other tissues, was finally found. Compared with the control group, EFEMP1 gene was upregulated in ocular tissues of patients with AMD, as well as in patients with pre-AMD, dry AMD, and wet AMD. Furthermore, the expression of EFEMP1 in wet AMD eyes was higher than that in patients with pre-AMD and dry AMD, but there was no significant difference between pre-AMD and dry AMD patients. (Figure 1; Table 2).

**TABLE 2:** EFEMP1 gene expression data in ocular tissues of patients with age-related macular degeneration (AMD) and healthy controls from GSE29801.

EFEMP1	AMD			
	Pre-AMD (n = 16)	Dry AMD (n = 16)	Wet AMD (n = 9)	Total (n = 41)
Control (n = 42)	*p* = 0.0229	*p* = 0.0003	*p* < 0.0001	*p* < 0.0001
Pre-AMD	—	*p* = 0.1295	*p* = 0.0005	—
Dry AMD	—	—	*p* = 0.0059	—

### Serum Fibulin-3 Level Increases in Patients With Wet Age-Related Macular Degeneration

**FIGURE 2:**
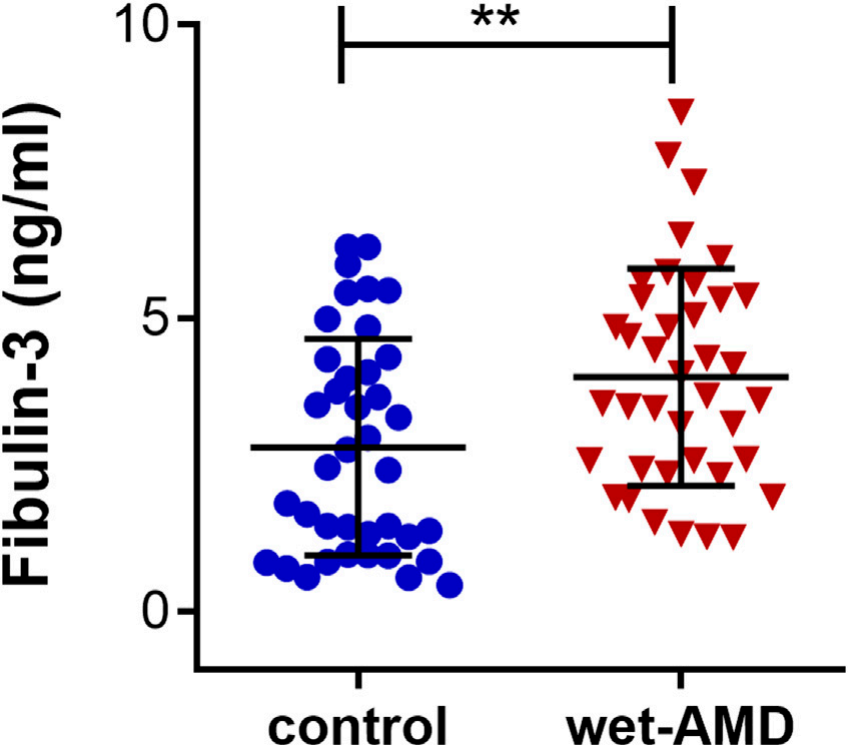
Serum fibulin-3 levels in wet AMD-affected patients and controls. Fibulin-3 serum concentration in wet AMD-affected patients was significantly higher than that in the controls. **p < 0.01.

To validate whether the serum concentration of EFEMP1 coding protein fibulin-3 was also upregulated in patients with wet AMD, serum samples from 39 wet AMD patients and 39 age- and gender-matched cataract controls were collected for ELISA. The mean levels of fibulin-3 in serum from patients with wet AMD and controls were 3.989 ± 1.852 ng/ml and 2.793 ± 1.847 ng/ml, respectively. There was a statistically significant increase in circulating fibulin-3 levels in patients with wet AMD compared with those in controls (*p* = 0.0056, Figure 2).

### Effects of EFEMP1 on Human Umbilical Vein Endothelial Cell Tube Formation

**FIGURE 3:**
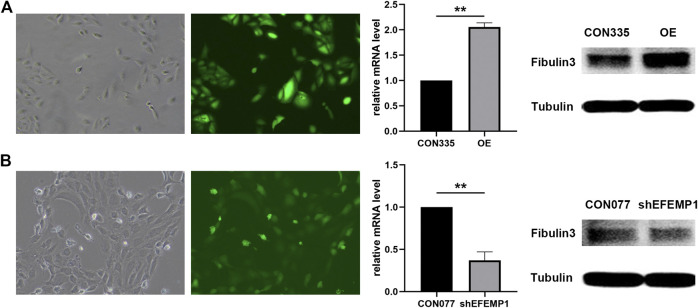
**(A)** EFEMP1 overexpressing (EFEMP1-OE) and **(B)** EFEMP1 knockdown HUVEC establishment and verification. High-content imaging visualization of HUVECs transfected with GFP-labelled GV492-EFEMP1/GV248-shEFEMP1 vectors was shown. qRT-PCR results of EFEMP1 relative mRNA levels showed a significant increase in GV492-EFEMP1-transfected HUVECs compared with that in GV492-CON335-transfected HUVECs and a significant decrease in GV248-shEFEMP1-transfected HUVECS compared with that in GV492-CON077-transfected HUVECs (**p < 0.01). EFEMP1 protein levels in the four HUVEC groups were tested using western blotting. EFEMP1 protein expression in GV492-EFEMP1-transfected HUVECs increased significantly compared with that in control CON335 and decreased in GV248-shEFEMP1-transfected HUVECS compared with that in control CON077 (**p < 0.01).

**FIGURE 4:**
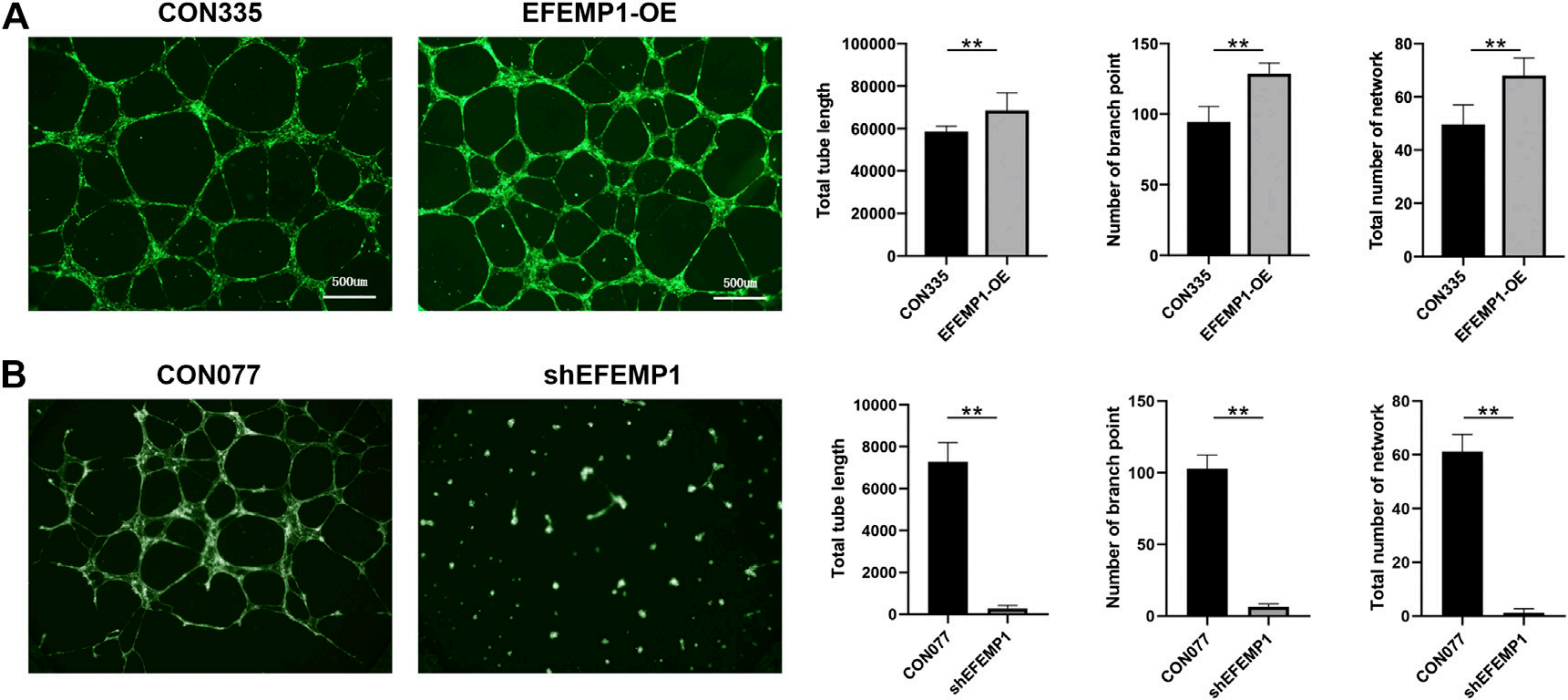
Effects of EFEMP1 on HUVEC tube formation. **(A)** Control CON335 HUVECs, EFEMP1-OE HUVECs, **(B)** control CON077 HUVECs, and shEFEMP1 HUVECs seeded on Matrigel were allowed to form tubes for 12 h in the absence of any additional stimulation. Total tube length, number of branch points, and total number of networks in captured images were measured using MetaMorph. The data represent the average of five independent replicates and the experiments were performed three times. **p < 0.01.

EFEMP1-OE and EFEMP1 knockdown (shEFEMP1) HUVECs were constructed (Figure 3). Next, we employed *in vitro* HUVEC tube formation assays to directly determine the effect of EFEMP1 on angiogenesis. In the absence of any additional stimulation in ECM with growth factor-reduced Matrigel, EFEMP1-OE HUVECS exhibited significantly enhanced tube formation activities, with increased mean HUVEC tube length, number of branch points, and total number of networks compared with those of the control CON335 HUVECs (Figure 4A, *p* < 0.01). In contrast, shEFEMP1 HUVECs exhibited markedly decreased tube formation capacities, with shorter mean tube length, fewer branch points, and smaller networks than those of the CON077 HUVECs (Figure 4B).

### Effects of EFEMP1 on Human Umbilical Vein Endothelial Cell Proliferation

**FIGURE 5:**
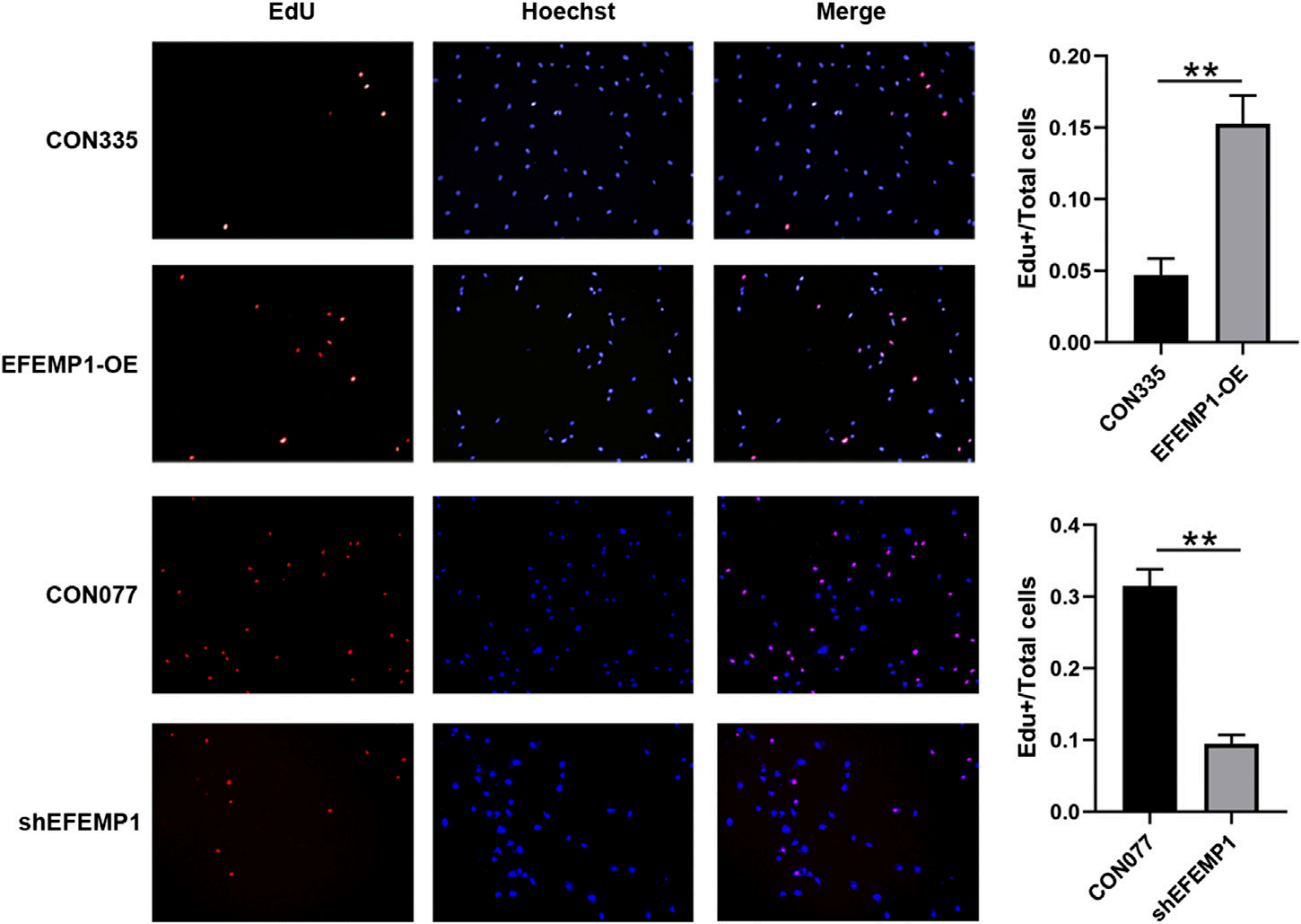
Effects of EFEMP1 on HUVEC proliferation. CON335 HUVECs, EFEMP1-OE HUVECs, CON077 HUVECs, and shEFEMP1 HUVECs were stained using EdU and Hoechst. EdU-positive cells were highlighted in red and nuclei in blue, following Hoechst staining. Proliferation capacities were analyzed by calculating the percentage of EdU-positive cells. Three independent experiments were performed, and five fields were randomly selected for statistical analysis in each experiment. **p < 0.01.

The proliferation of EFEMP1-OE HUVECs increased significantly compared to that of CON335 HUVECs, whereas the proliferation of shEFEMP1 HUVECs decreased significantly compared to that of CON077 HUVECs (Figure 5). This indicated that fibulin-3 played an important role in promoting cell proliferation in HUVECs.

### Origin of the Proangiogenesis Property of EFEMP1

**FIGURE 6:**
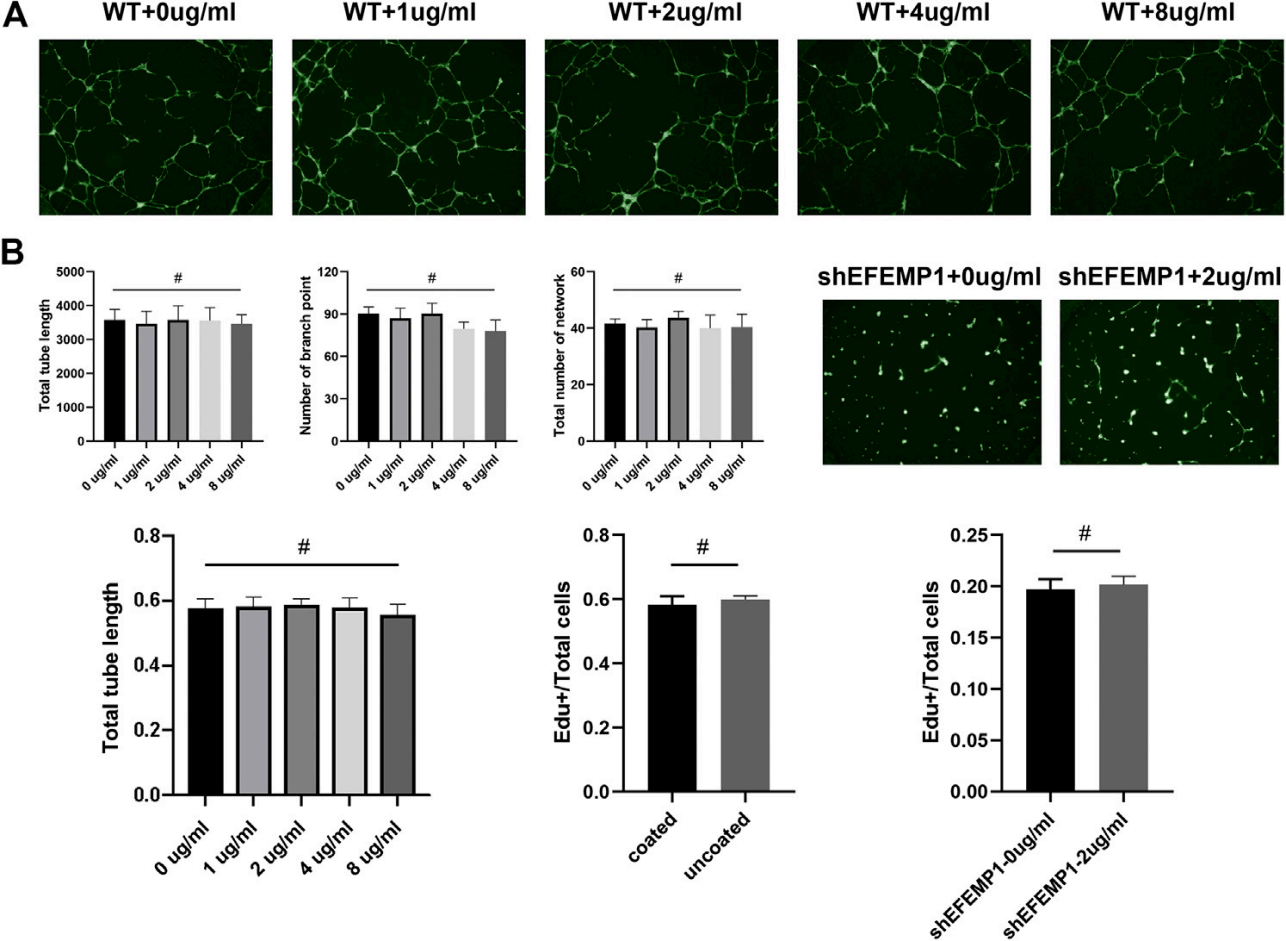
Origin of the proangiogenesis property of EFEMP1. **(A)** Tube formation of wild type (WT) HUVECs treated with different concentrations (0 µg/ml, 1 µg/ml, 2 µg/ml, 4 µg/ml, and 8 µg/ml) of recombinant human fibulin-3 protein and shEFEMP1 HUVECs treated with or without fibulin-3 protein was tested. **(B)** Proliferation of wild type (WT) HUVECs treated with different concentrations (0 µg/ml, 1 µg/ml, 2 µg/ml, 4 µg/ml, and 8 µg/ml) of recombinant human fibulin-3 protein, WT HUVECs in fibulin-3 coated or uncoated plates, and shEFEMP1 HUVECs treated with or without fibulin-3 protein was tested using EdU assay. ^#^ *p* > 0.05.

EFEMP1 is a member of the secreted extracellular glycoprotein family, and its overexpression increases both the intracellular and extracellular fibulin-3 levels. Therefore, to reveal whether the origin of the proangiogenesis property of EFEMP1 is extracellular or intracellular, the effects of extracellular fibulin-3 on wildtype (WT) and shEFEMP1 HUVECs were examined. It seemed that WT HUVECS treated with different concentrations of recombinant human fibulin-3 protein exhibited similar tube formation capacities and no significant differences in mean tube length, number of branch points, and total number of networks were found among groups. In addition, the addition of fibulin-3 in ECM could not recover the inhibited tube formation capacity of shEFEMP1 HUVECs (Figure 6A). Similarly, the proliferation of WT HUVECs was not affected by different concentrations of extracellular fibulin-3, and no significant differences in proliferation capacities were found between WT HUVECs in fibulin-3 coated or uncoated plates (Figure 6B). Moreover, the proliferation of shEFEMP1 HUVECs was not increased by the addition of extracellular fibulin-3 (Figure 6B). The results indicated that intracellular, but not extracellular, increase in fibulin-3 could promote angiogenesis in HUVECs.

### Effects of EFEMP1 on the Expression of Vascular Endothelial Growth Factor in Human Umbilical Vein Endothelial Cell

**FIGURE 7:**
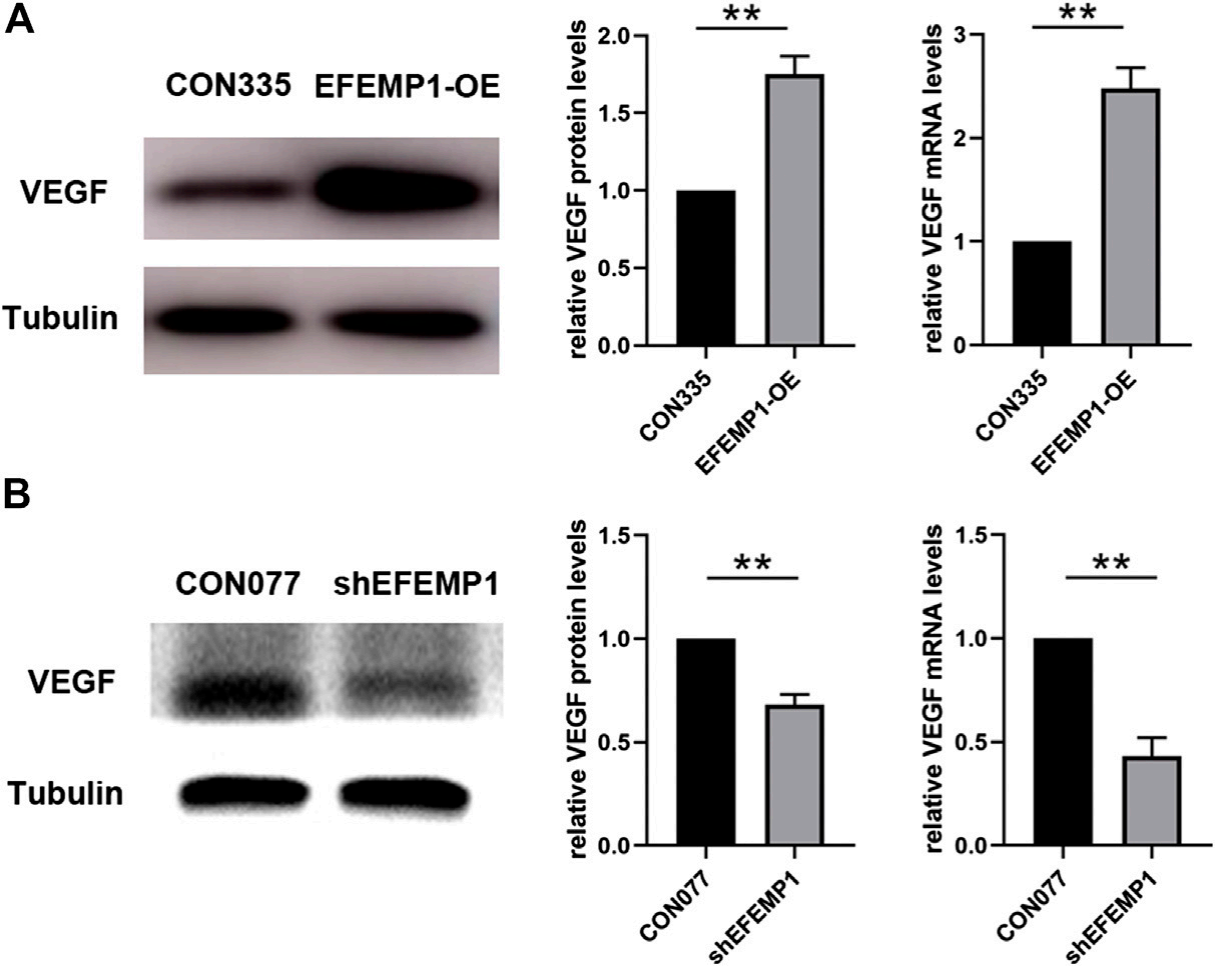
Effects of EFEMP1 on VEGF expression. VEGF mRNA and protein level alterations in **(A)** CON335 HUVECs, EFEMP1-OE HUVECs, **(B)** CON077 HUVECs, and shEFEMP1 HUVECs were detected using qRT-PCR and western blotting, respectively. **p < 0.01.

As shown in Figure 7, VEGF mRNA and protein levels were significantly increased by overexpression of fibulin-3, whereas knockdown of fibulin-3 inhibited the expression of VEGF at the mRNA and protein levels. This indicated that VEGF signaling may participate in the proangiogenesis process induced by fibulin-3. These results are also consistent with the findings in a previous study whereby CNV cases with R345W mutation in EFEMP1 were sensitive to anti-VEGF treatment (Sohn et al., 2011).

## Discussion

AMD is a disease with complex inheritance and epigenetic changes (DeAngelis et al., 2017). CNV development is a common and sight-threatening complication. Based on numerous genetic studies of AMD, approximately 50% of the heritability of AMD can be explained by two major loci harboring coding and noncoding variation at chromosomes 1q (CFH) and 10q (ARMS2/HTRA1) (Klein et al., 2005; Hageman et al., 2005; Rivera et al., 2005; Deangelis et al., 2008). Recently, a large sample genome-wide association study (GWAS) has highlighted new genes and pathways in the development of AMD, including complement activation, collagen synthesis, lipid metabolism/cholesterol transport, endodermal cell differentiation, and extracellular matrix organization, indicating that many unknown genetic changes remain to be discovered in the initiation and development of AMD (Fritsche et al., 2016). Compared to previous GWAS studies, gene expression profiling of human ocular tissues has great potential to identify gene coexpression modules, to build molecular models with predictive utility, and to elucidate functional networks of AMD (Newman et al., 2012). In the present study, we aimed to screen novel biomarkers from the perspective of transcriptome profiling of AMD patients’ RPE-choroid and retina tissue samples (GSE29801), using integrated bioinformatic analysis, verify its expression in peripheral blood, and elucidate its possible biological function. To our knowledge, no similar studies have been conducted previously.

In this study, we downloaded the RNA sequencing microarray data from the GEO database. NCBI-GEO is a free database for microarray/gene profiling and next-generation sequencing. Furthermore, we analyzed the transcriptome of differentially expressed genes in ocular tissues derived from AMD donor patients compared with normal donor controls. As a result, the differentially upregulated EFEMP1 gene was screened.

It is generally believed that the human EFEMP1 cDNA encodes a secreted extracellular protein named fibulin-3, which consists of 493 amino acids with a predicted molecular weight of 55 kDa (Lecka-Czernik et al., 1995). Fibulin-3 is one of seven highly conserved members of the fibulin family of extracellular matrix (ECM) proteins (Zhang and Marmorstein, 2010), which is implicated in extracellular matrix remodeling, cell proliferation, and organogenesis (Chen et al., 2018). Although EFEMP1 is a broadly expressed gene throughout the body (Marmorstein et al., 2002; Kobayashi et al., 2007; Zhang and Marmorstein, 2010), its expression in the retina is significantly higher than in other tissues (Figure 1). A missense mutation R345W was found to be associated with Malattia Leventinese/Doyne honeycomb retinal dystrophy (ML/DHRD) (Stone et al., 1999). In normal eyes, EFEMP1 accumulated predominantly in the nerve fiber layer and the photoreceptor inner and outer segments; less intense accumulation was observed in the outer nuclear layer (ONL) and the inner (IPL) and outer (OPL) plexiform layers. No accumulation was observed in the RPE, Bruch’s membrane, the choroid (CH), or the inner nuclear layer (INL). Nevertheless, in both ML and AMD eyes, abnormally accumulated EFEMP1 is seen beneath the RPE overlaying sub-RPE deposits and accelerates the process of drusen formation (Marmorstein et al., 2002). Recent studies have shown that EFEMP1 knockout has protective effects on the progress of sub-RPE deposits in mouse eyes (Stanton et al., 2017), suggesting that the existence of EFEMP1 may be required for sub-RPE deposit formation. Nevertheless, the association between EFEMP1 and wet AMD has not been reported thus far, and the function of EFEMP1 in CNV, especially in vascular endothelial cells, is still unclear.

According to the present study, the expression of EFEMP1 was upregulated in the retinal-choroid tissue of both dry AMD and wet AMD patients. Moreover, the increase in wet AMD was more significant than that in dry AMD. We also described higher levels of serum fibulin-3 in wet AMD compared to controls. This may emphasize an important role of EFEMP1 in the advanced CNV stage of the disease, and not only in the early drusen formation phase, indicating that EFEMP1 might be a novel biomarker of wet AMD that could be detected through peripheral blood examination.

Recently, an increasing number of studies have implicated that EFEMP1 plays an important but contradictory role in regulating angiogenesis, as it appears to have different functions in different tissues. In lung, liver, breast, ovarian, and prostate cancers, fibulin-3 acts as an angiogenesis antagonist (Sadr-Nabavi et al., 2009; Hu et al., 2011; Chen et al., 2013). Downregulation of fibulin-3 results in tumor angiogenesis (Albig et al., 2006). However, in cervical carcinoma, pancreatic cancer, high-grade gliomas, and psoriasis, overexpressed fibulin-3 was shown to upregulate VEGF expression and induce angiogenesis (Seeliger et al., 2009; Song et al., 2011; Nandhu et al., 2014; Wang et al., 2019). This property may be correlated with its context-specific manner (Albig et al., 2006). However, its role in the development of ocular angiogenesis has not been investigated. The higher EFEMP1 expression level in the retinal-choroid tissues with CNV lesions was demonstrated in the current study, indicating that EFEMP1 plays a proangiogenic role in wet AMD eyes. The current study also highlights the active role of EFEMP1 in HUVECs *in vitro*. We found that overexpression of EFEMP1 promoted tube formation and cell proliferation of HUVECs, whereas knockdown of EFEMP1 inhibited tube formation and proliferation capacities. One possible explanation for these results is the change in extracellular fibulin-3 levels, which was caused by the increase or decrease in fibulin-3 secretion in HUVECs transfected with fibulin-3 cDNA or shRNA. Indeed, higher extracellular fibulin-3 levels secreted by tumor cells could induce proangiogenic behavior in endothelial cells (Nandhu et al., 2014; Wang et al., 2019). Therefore, we reexamined the angiogenic behavior of HUVECs using highly purified fibulin-3. Remarkably, extracellular fibulin-3 was insufficient to increase HUVEC tube formation and proliferation. Moreover, tubulogenesis and proliferation inhibited by shEFEMP1 was not recovered in the presence of additional fibulin-3. Taken together, these results suggest that the proangiogenic effects of EFEMP1 in HUVECs are, at least in part, dependent on intracellular modulation. In addition, the expression of VEGF in HUVECs was upregulated by EFEMP1 overexpression and downregulated by EFEMP1 knockout, providing indirect evidence that the VEGF signaling pathway may participate in the proangiogenesis of EFEMP1.

Although R345W mutations have been reported in ML, no mutation in EFEMP1 has been reported to be associated with AMD (Stone et al., 1999). In the absence of a mutation, it is possible that modifications due to aging, cigarette smoking, or oxidative, thermal, or other stress cause overexpression of EFEMP1 (Giasson et al., 2000; Stanton et al., 2017) in RPE cells and vascular endothelial cells in the retina of AMD eyes. On one hand, secreted fibulin-3 could partially drain into the blood through disrupted blood–retina barriers, resulting in an increase in serum fibulin-3 levels. On the other hand, increased fibulin-3 may promote angiogenesis, which is common in tumor angiogenesis. In the present study, upregulated ocular expression of EFEMP1 and increased serum level of fibulin-3 have been verified in patients with wet AMD. We also demonstrated that HUVECs-origin fibulin-3 contributes to endothelial cell tube formation, proliferation, and VEGF production. Although extracellular matrix origin fibulin-3 promotes sub-RPE deposits and accelerates the process of drusen formation (Marmorstein et al., 2002; Stanton et al., 2017), the proangiogenic properties of EFEMP1 are of endothelium origin according to the present study. The angiogenesis modulation mechanism by fibulin-3 needs to be studied in more detail through both *in vivo* and *in vitro* experiments in the future. One possible explanation would be that, as an extracellular glycoprotein, fibulin-3 is translated into the endoplasmic reticulum, where it is folded and processed before transport to the Golgi and eventually secreted. Overexpression of fibulin-3 and its accumulation in the endoplasmic reticulum could lead to the activation of the unfolded protein response on demand and further induce transcriptional upregulation of VEGF, which has been reported in RPE cells (Roybal et al., 2005).

The current study is interesting owing to two principal reasons. First, we revealed for the first time that EFEMP1 gene expression is upregulated in wet AMD eyes, which could be detected through serum examination, providing new molecular biomarkers and gene expression signatures of AMD. Second, we discovered that EFEMP1 could promote angiogenesis of endothelial cells to foster CNV development in the eye, providing new insights into the landscape of AMD pathophysiology and new targets for CNV treatment. This study also has some limitations and shortcomings. First, the sample size, especially in the wet AMD group in the current study, was limited; therefore, the results of this study are preliminary and larger sample sizes are needed to produce a solid confirmation. Second, only *in vitro* experiments were conducted in the present study; thus, further *in vivo* experiments are needed to provide more direct evidence of the role of EFEMP1 in CNV formation.

## Conclusion

In summary, we have demonstrated several important findings. First, an increase in EFEMP1 expression was observed in the retinal-choroid tissues of eyes with AMD, which was more significant in wet AMD than in dry AMD. Second, higher levels of serum fibulin-3 were detected in wet AMD. Finally, cell-origin EFEMP1 substantially promoted tube formation, enhanced cell proliferation, and increased the expression of VEGF in HUVECs. These results may offer novel insights into AMD pathogenesis and represent new targets for the development of AMD-directed therapeutics and diagnostics, but they need further confirmation by *in vivo* experiments.

## Data Availability Statement

The original contributions presented in the study are included in the article/Supplementary Material, further inquiries can be directed to the corresponding authors.

## Ethics Statement

The studies involving human participants were reviewed and approved by the Medical Ethics Committee at the Shanghai General Hospital affiliated to the Shanghai Jiao Tong University (No.2016KY115-2). The patients/participants provided their written informed consent to participate in this study.

## Author Contributions

LC and CC conceived and coordinated the project, enrolled participants, collected samples, and performed serological analysis, complied the figures and the tables, and wrote the manuscript. WG performed cell experiments and statistical analysis of all data and compiled functional enrichment analysis and protein–protein interaction network analysis. KL helped to collect participants and coordinate data analysis. QZ and PL helped with data curation and searching information in different databases. FY sourced the database and screened the hub genes. XX conceived and directed the project and wrote the manuscript. All authors reviewed the manuscript.

## Funding

This study was supported by the National Key R&D Program of China (2016YFC0904800, 2019YFC0840607); National Science and Technology Major Project of China (2017ZX09304010); National Natural Science Foundation of China (81800831, 81800835, and 82000880); Shanghai Sailing Program (18YF1419700 and 19YF1439400); Shanghai “Rising Stars of Medical Talent” Youth Development Program (Youth Medical Talents Specialist Program), and Cross-Research Foundation of Shanghai Jiaotong University (ZH2018QNA13). No author has a financial or proprietary interest in any material or method mentioned.

## Conflict of Interest

The authors declare that the research was conducted in the absence of any commercial or financial relationships that could be construed as a potential conflict of interest.
